# Photoinduced Phase Transitions of Imine-Based Liquid Crystal Dimers with Twist–Bend Nematic Phases

**DOI:** 10.3390/ma17133278

**Published:** 2024-07-03

**Authors:** Yuki Arakawa, Yuto Arai

**Affiliations:** Department of Applied Chemistry and Life Science, Graduate School of Engineering, Toyohashi University of Technology, 1-1 Hibarigaoka, Tempaku-cho, Toyohashi 441-8580, Aichi, Japan; arai.yuto.du@tut.jp

**Keywords:** liquid crystal dimer, twist–bend nematic phase, thioether, ester direction, imine bond, benzylideneaniline, photoinduced phase transition

## Abstract

Photoisomerizable molecules in liquid crystals (LCs) allow for photoinduced phase transitions, facilitating applications in a wide variety of photoresponsive materials. In contrast to the widely investigated azobenzene structure, research on the photoinduced phase-transition behavior of imine-based LCs is considerably limited. We herein report the thermal and photoinduced phase-transition behaviors of photoisomerizable imine-based LC dimers with twist–bend nematic (N_TB_) phases. We synthesize two homologous series of ester- and thioether-linked *N*-(4-cyanobenzylidene)aniline-based bent-shaped LC dimers with an even number of carbon atoms (*n* = 2, 4, 6, 8, and 10) in the central alkylene spacers, namely, CBCOO*n*SBA(CN) and CBOCO*n*SBA(CN), possessing oppositely directed ester linkages, C=OO and OC=O, respectively. Their thermal phase-transition behavior is examined using polarizing optical microscopy and differential scanning calorimetry. All dimers form a monotropic N_TB_ phase below the temperature of the conventional nematic (N) phase upon cooling. Remarkably, the N_TB_ phases of CBCOO*n*SBA(CN) (*n* = 2, 4, 6, and 8) and CBOCO*n*SBA(CN) (*n* = 6 and 8) supercool to room temperature and vitrify without crystallization. In addition, the phase-transition temperatures and entropy changes of CBCOO*n*SBA(CN) are lower than those of CBOCO*n*SBA(CN) at the same *n*. Under UV light irradiation, the N_TB_ and N phases transition to the N and isotropic phases, respectively, and reversibly return to their initial LC phases when the UV light is turned off.

## 1. Introduction

Photoisomerizable building blocks coupled with liquid crystals (LCs) have attracted considerable attention for a variety of applications such as optical storage [1,2,3,4], photomobile materials [5,6,7,8], photoalignments [9,10], and adhesion [11,12]. In particular, the azobenzene (Ph–N=N–Ph) structure is well-known for its mesogenic and reversible *trans*–*cis* isomerization abilities. The *trans* isomer, stable under ambient temperature, transforms into the *cis* isomer upon UV light irradiation [13], and the generated *cis* isomer reverts to the initial *trans* isomer upon irradiation with visible light or by heating. This *trans*-to-*cis* isomerization disorders the molecular arrangements in LC phases. Consequently, LC phases, such as nematic (N) [1,14], blue (BP) [15], layered smectic (Sm) [16], and columnar phases, [17] in *trans* azobenzenes transition to isotropic (Iso) phases, which reversibly return to the initial LC phases. However, some LC phases rearrange into other LC and chiral Iso phases [18,19,20,21]. Even solid crystals of certain azobenzenes can liquefy to the Iso phase [11,22]. These phenomena are known as the photoinduced (or light-induced) phase transitions for LCs. Additionally, photoisomerization of the azobenzene structures follows the Weigert effect. Upon irradiation with linearly polarized light, the molecular anisotropic polarization direction (long axis) of azobenzene becomes normal to that of the exposed light, thus inducing a change in the shape and surface of LC materials for the applications mentioned above.

Similarly, the rigid and anisotropic imine-based *N*-benzylideneaniline (Ph–CH=N–Ph) structure is a commonly used building block for LC molecules [23,24,25,26,27,28,29,30,31,32,33,34,35,36,37,38,39,40,41,42,43]. Similar to that of azobenzene, the –CH=N– imine bond in the *N*-benzylideneaniline structure undergoes reversible *trans*–*cis* isomerization upon irradiation with UV light [44,45,46]. The *trans*-to-*cis* isomerization of the imine bond requires more energy (~50–60 kcal mol^−1^) [46,47,48] than that of azobenzene (<~50 kcal mol^−1^) [7]. As a notable difference, the imine bond shows remarkably faster thermal back-relaxation from *cis*-to-*trans* forms than the azo bond. The energy barrier for the thermal back-relaxation of imine bonds (~16 kcal mol^−1^) is lower than those of azobenzene (~23 kcal mol^−1^) and structurally similar stilbene Ph–CH=CH–Ph (42 kcal mol^−1^), resulting in a short half-life of the *cis* isomer of the imine at ambient temperature [45,46]. Accordingly, the *cis* state of imine molecules can be detected by UV–visible spectroscopy only at low temperatures below –70 °C and not at ambient temperatures [45,46]. An advantage of *N*-benzylideneaniline over azobenzene is its transparency in the visible region, which is crucial for practical applications. However, the current research on the photoisomerization behaviors of *N*-benzylideneaniline-based LCs is considerably limited [49,50,51,52,53,54]. In the presence of *N*-benzylideneaniline-based molecules, the LC phases reversibly transition to Iso phases upon UV light irradiation [53,54]. Kawatsuki and Kondo et al. investigated the photoisomerization of *N*-benzylideneaniline-based LC polymers for photoreorientation and adhesion applications [49,50,51,52,53]. Hu and Yu recently reported a solid-to-liquid phase transition of *N*-benzylideneaniline with asymmetric alkyl chain substitution [54]. In addition to the photoisomerizability, Terentjev et al. demonstrated the applicability of the bond exchangeable ability of *N*-benzylideneaniline embedded in LC elastomers [55]. Therefore, the use of *N*-benzylideneaniline for LCs has been continuously increasing.

Over the last decade, the helical twist–bend nematic (N_TB_) phase for bent molecules has gained widespread interest in LC science [56,57,58]. The N_TB_ phase exhibits heliconical structures with pitches in the range of several to tens of nanometers, generated by the precession of bent molecules [59,60,61]. The heliconical structures of the N_TB_ phase form pseudo-layers, making the physical properties of this phase more similar to those of layered Sm phases than to the conventional N phase [62,63,64,65]. The majority of the N_TB_ phase is observed for bent LC dimers composed of two mesogenic arms linked via a central spacer with an odd number of total atoms along the spacer [66,67,68,69,70,71]. The molecular biaxialities of the bent dimers can be associated with N_TB_ phase induction [72]. Twist–bend nematogenic bent dimers are also useful for photonic applications with wavelength or color tunability [73,74]. The photoinduced phase-transition behavior of the N_TB_ phase has been evaluated for azobenzene-based LC dimers [75,76]. Under moderate UV irradiation, the N_TB_ phase transitions to the conventional N phase, exhibiting reversibility when the light is switched on and off [75]. Owing to the Weigert effect, the molecular and helical axes of the N_TB_ phase can be oriented perpendicular to the polarization direction of the polarized linear light [76]. However, to the best of our knowledge, the photoinduced phase-transition behaviors of the LC phases for imine-based LC dimers have not been reported to date.

**Figure 1:**
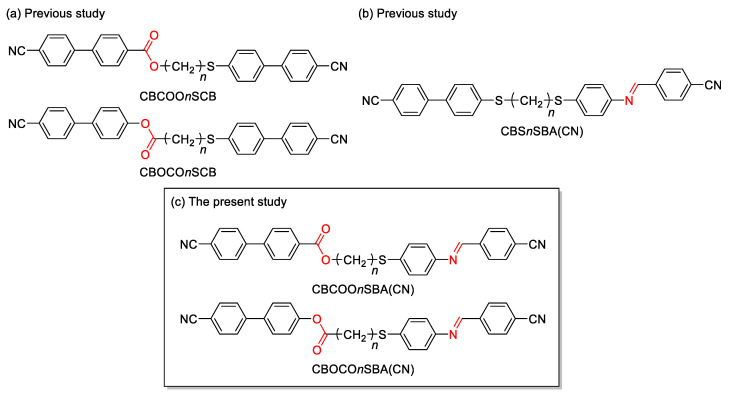
Molecular structures of (**a**) CBCOO*n*SCB, CBOCO*n*SCB, [33,77] (**b**) CBS*n*SBA(CN) [34] from our previous study, and (**c**) CBCOO*n*SBA(CN) and CBOCO*n*SBA(CN) in the present study.

We herein report the synthesis and thermal and photoinduced phase-transition behaviors for the N and N_TB_ phases of *N*-benzylideneaniline-based LC dimers. We synthesized two new homologous series of oppositely directed ester- and thioether-linked *N*-(4-cyanobenzylidene)aniline- and 4-cyanobiphenyl-based dimers [CBCOO*n*SBA(CN) and CBOCO*n*SBA(CN)] with even-numbered alkylene spacers (*n* = 2, 4, 6, 8, and 10) and total odd number of atoms to obtain bent molecular shapes (Figure 1c). The series differs in the ester linkage directions, viz., C=OO or OC=O for CBCOO*n*SBA(CN) and CBOCO*n*SBA(CN), respectively. We previously reported two homologous series of oppositely directed ester- and thioether-linked cyanobiphenyl LC dimers, CBCOO*n*SCB and CBOCO*n*SCB, as given in Figure 1a [33,77]. These dimers exhibit the N_TB_ phases, which can be stably cooled to the ambient temperature and vitrified; however, their phase-transition behaviors, helical pitches, and viscoelastic properties differ substantially owing to the ester directions [33,77,78,79]. Furthermore, thioether-linked *N*-(4-cyanobenzylidene)aniline- and 4-cyanobiphenyl-based bent dimers, CBS*n*SBA(CN) (Figure 1b) exhibit stable and vitrifiable N_TB_ phases [34]. In this context, we designed *N*-(4-cyanobenzylidene)aniline-containing LC dimers with thioether and oppositely directed ester linkages for new twist–bend nematogens to elucidate their thermal and photoinduced phase-transition behaviors. The thermal phase-transition behavior was evaluated using polarizing optical microscopy (POM) and differential scanning calorimetry (DSC). Additionally, POM was used to analyze the photoinduced phase transitions via UV irradiation of the LC phases.

## 2. Materials and Methods

**Scheme 1:**
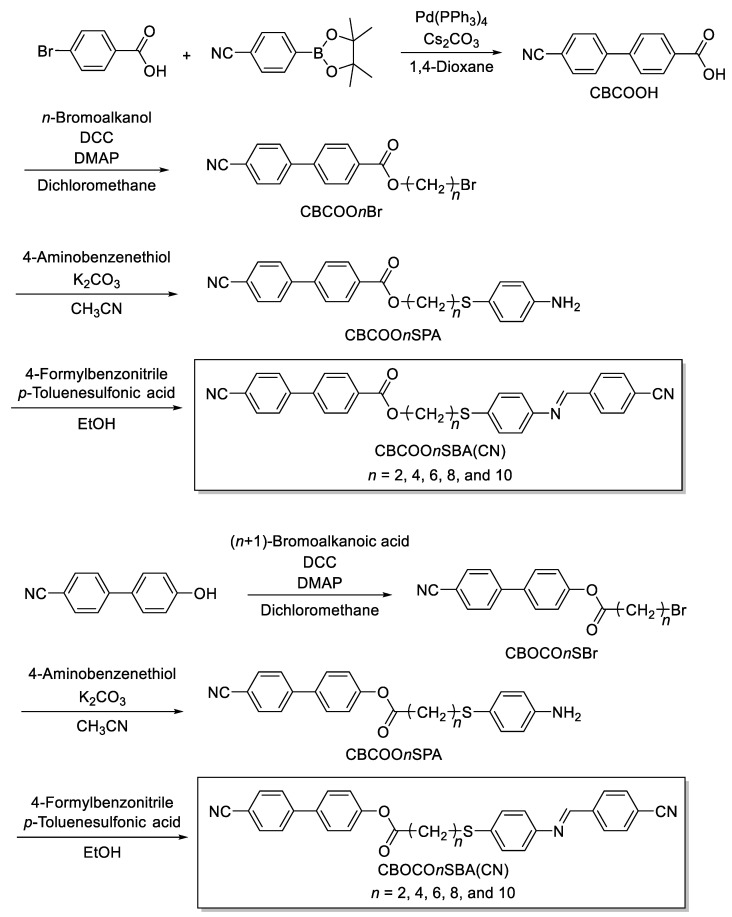
Synthesis of CBCOO*n*SBA(CN) and CBOCO*n*SBA(CN), where *n* = 2, 4, 6, 8, and 10.

All chemicals were commercially available and used as received. The synthetic scheme for the two homologous series is outlined in Scheme 1. The molecular structures were characterized by ^1^H and ^13^C nuclear magnetic resonance (NMR) spectroscopies using JEOL JNM-ECS400 (400 and 100 MHz for ^1^H and ^13^C NMR, respectively) or JEOL JNM-ECX500 (500 and 125 MHz for ^1^H and ^13^C NMR, respectively) spectrometers. The synthetic procedures and characterization data are provided in Supporting Information. The thermal phase transitions and phase identification were evaluated by POM using an Olympus BX50 optical microscope equipped with a Linkam temperature controller (LK-600PM). For planar and uniaxial alignment observation, polyimide surface cells with thicknesses of 1 and 7 µm were obtained from EHC Co., Ltd. (Hachioji-shi, Japan). The phase-transition temperatures and associated enthalpy changes were determined using a Shimadzu DSC-60 Plus at a rate of 10 °C min^−1^ under a nitrogen gas flow rate of 50 mL min^−1^ and liquid nitrogen (Liq. N_2_) was added for cooling. Indium was used for calibration. The photoinduced phase-transition behavior was observed using POM upon UV light irradiation. The 365-nm UV light from the outer light source (Asahi Spectra LAX-C100) was reflected by a dichroic half mirror inserted in an Olympus BX53M optical microscope, which was exposed to the LC samples in the non-treated glass cells with two pieces of commercial glass on a Mettler Toledo HS82 hot-stage system. The UV light intensity (approximately 3 mW cm^−2^) was monitored at each sample height using an Ushio UIT-201 digital UV intensity meter equipped with an Ushio photodetector UVD-365PD. The UV–visible absorption spectra for both homologous series with *n* = 6 were recorded in tetrahydrofuran (THF) using a JASCO V-630 UV–visible spectrophotometer.

## 3. Results and Discussion

### 3.1. Thermal Phase Transitions

**Figure 2:**
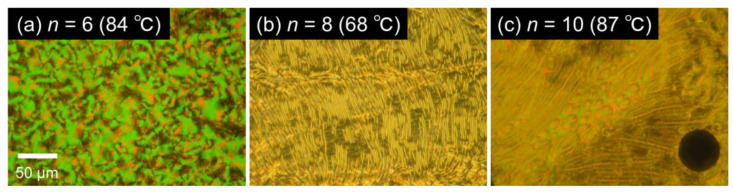
Optical textures observed in the N_TB_ phases in a non-treated glass cell upon cooling CBCOO*n*SBA(CN): (**a**) blocky texture for *n* = 6, (**b**) striped texture for *n* = 8, and (**c**) rope-like and focal-conic-like textures for *n* = 10.

The melting temperatures (*T*_m_) upon heating, Iso–N phase transition (*T*_IN_), N–N_TB_ phase-transition (*T*_NNTB_), glass transition (*T*_g_), and crystallization (*T*_Cr_) temperatures upon cooling, along with their associated enthalpy changes (Δ*H*) for CBCOO*n*SBA(CN) are listed in Table 1. Upon heating, the shorter spacer CBCOO*n*SBA(CN) homologs (*n* = 2, 4, and 6) did not exhibit LC phases, whereas longer spacers (*n* = 8 and 10) formed the conventional N phase. All the CBCOO*n*SBA(CN) homologs exhibited N_TB_ and N phases upon cooling. The N_TB_ phase was identified by blocky (Figure 2a), striped (Figure 2b), focal-conic-like, or rope-like (Figure 2c) textures in the non-treated glass cells as shown for *n* = 6, 8, and 10, respectively. Specifically, blocky textures were often observed beneath the N–N_TB_ phase transition, as shown in Figure 2a. Shearing the cells led to striped textures along the shear direction, as shown in Figure 2b. This behavior is attributed to the Helfrich–Hurault mechanism of the pseudo-layered N_TB_ phase [63,80].

**Figure 3:**
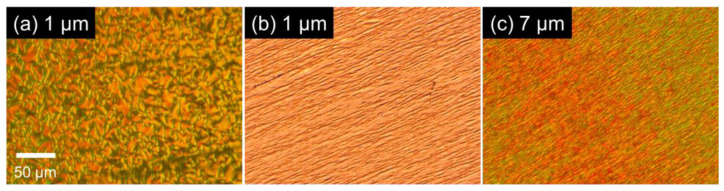
Optical textures of the N_TB_ phase of CBCOO2SBA(CN) in (**a**) non-treated and uniaxially rubbed planar alignment glasses with cell thicknesses of (**b**) 1 and (**c**) 7 µm.

The optical texture of the N_TB_ phase of CBCOO2SBA(CN) was not as regular in the non-treated glass cells, as shown in Figure 3a. POM using uniaxially rubbed planar alignment cells with 1- and 7-µm thicknesses show lines or thin stripes growing along the rubbing direction, as shown in Figure 3b,c, respectively. The stripes for a 7-µm-thick cell were wider and clearer than those for a 1-µm-thick one, which could be attributed to the Helfrich–Hurault-like instability of the pseudo-layer nature of the N_TB_ phase [80]. The stripes of CBCOO2SBA(CN) were relatively thinner and more ambiguous than those of typical stripes, which could be attributed to its high viscoelastic properties, as observed for the structurally similar cyanobiphenyl dimer CBCOO4SCB, exhibiting similar textural behaviors [79].

**Figure 4:**
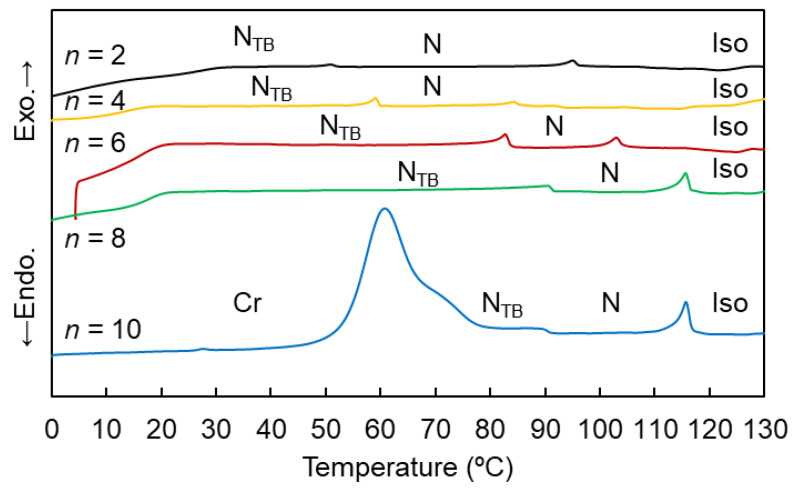
DSC curves of CBCOO*n*SBA(CN) upon cooling at a rate of 10 °C min^−1^.

The N–N_TB_ phase-transition peaks of all the CBCOO*n*SBA(CN) dimers were detected by DSC, as shown in Figure 4. The shapes of the N–N_TB_ phase-transition peaks suggest that they are first-order for shorter spacer CBCOO*n*SBA(CN) dimers (*n* < 8), which could be attributed to the strong molecular biaxiality (molecular bend) of these dimers owing to the small bond angle of the C–C(=O)–O ester linkage and the shorter spacer, as reported previously [33,77,78,79]. The rigidity of the C–C(=O)–O ester linkage may also help to enhance their molecular biaxiality for the shorter spacer in terms of narrower plausible conformation distributions.

**Figure 5:**
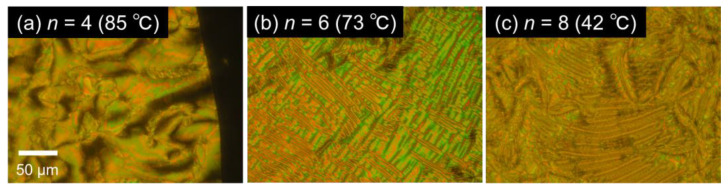
Optical textures of the N_TB_ phase of CBOCO*n*SBA(CN) in a non-treated glass cell upon cooling: (**a**) blocky texture for *n* = 4, (**b**) striped texture for *n* = 6, and (**c**) rope-like texture for *n* = 8. The blocky texture of (**a**) CBOCO4SBA(CN) was recorded in a supercooled region that did not crystallize.

**Figure 6:**
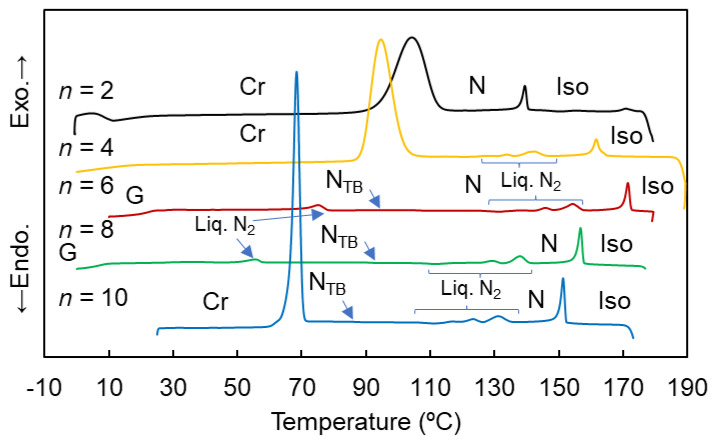
DSC curves of CBOCO*n*SBA(CN) upon cooling at a rate of 10 °C min^−1^.

The phase-transition results for CBOCO*n*SBA(CN) are listed in Table 2. Upon heating, the CBOCO*n*SBA(CN) homologs (*n* = 4, 6, 8, and 10) showed the N phase, whereas CBOCO2SBA(CN) did not exhibit the LC phase. All the homologs exhibited the N_TB_ and N phases upon cooling, similar to the CBCOO*n*SBA(CN) homologs. The N_TB_ phase was characterized by typical blocky, striped, and focal-conic-like and rope-like textures in the non-treated glass cells for *n* = 4, 6, and 8, respectively, as shown in Figure 5, which are similar to those of CBCOO*n*SBA(CN). Figure 6 shows the DSC curves for CBCOO*n*SBA(CN) upon cooling. The shorter-spacer CBOCO*n*SBA(CN) dimers (*n* = 2 and 4) crystallized from the conventional N phase during DSC, and the crystallization peaks cover the N–N_TB_ phase-transition peaks upon cooling in Figure 6. Therefore, their *T*_NNTB_ values were determined by POM. Figure 6 reveals the N_TB_ phase of CBOCO*n*SBA(CN) with *n* = 6 and 8 vitrified at approximately 10 °C. CBCOO10*n*SBA(CN) crystallized from the N_TB_ phase.

**Figure 7:**
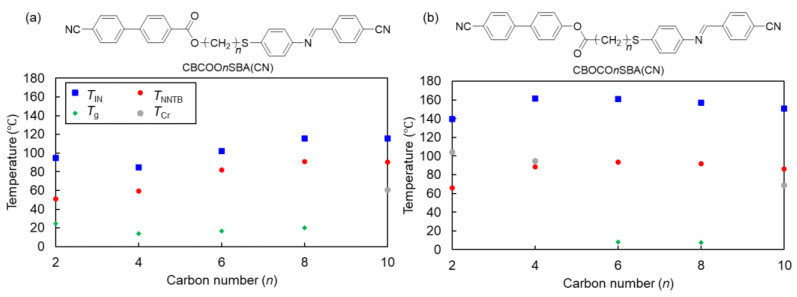
*T*_IN_, *T*_NNTB_, *T*_g_, and *T*_Cr_ values as a function of *n* for (**a**) CBCOO*n*SBA(CN) and (**b**) CBOCO*n*SBA(CN) upon cooling.

**Figure 8:**
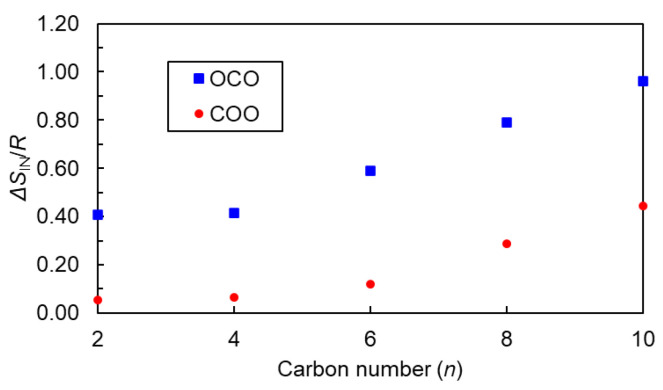
Δ*S*_IN_/R values as a function *n* for CBCOO*n*SBA(CN) (red circles) and CBOCO*n*SBA(CN) (blue squares) upon cooling.

The *T*_IN_, *T*_NNTB_, and *T*_g_ values of CBCOO*n*SBA(CN) and CBOCO*n*SBA(CN) upon cooling are plotted as a function of temperature in Figure 7a and Figure 7b, respectively. Notably, the *T*_IN_ values of CBCOO*n*SBA(CN) are remarkably lower than those of CBOCO*n*SBA(CN), which is similar to the difference in those of previously reported CBCOO*n*SCB and CBOCO*n*SCB [33,77]. This behavior could be attributed to a more bent molecular shape of CBCOO*n*SBA(CN) or a more anisotropic one of CBOCO*n*SBA(CN) [33,77]. The Ar–C–O bond angle in the COO ester linkage (109°) is smaller than that of Ar–O–C in the OCO-ester linkage, similar to Ar–O–C in the ether linkage. The smaller Ar–C–O bond angle results in a more bent molecular shape for CBCOO*n*SBA(CN) compared to that for CBOCO*n*SBA(CN). The more rigid COO ester than OCO facilitates the stronger biaxiality of CBCOO*n*SBA(CN) than that of CBOCO*n*SBA(CN) [33,77]. The entropy changes (Δ*S*_IN_/*R*) at the I–N phase transition upon cooling scaled by the gas constant (*R* = 8.31 J K^−1^ mol^−1^), which are indicators of the molecular biaxiality [81], are plotted as a function of *n* in Figure 8. The Δ*S*_IN_/*R* values are remarkably lower for CBCOO*n*SBA(CN) than that for CBOCO*n*SBA(CN), indicating the stronger biaxiality or considerably more molecular bending of CBCOO*n*SBA(CN).

In contrast, the *T*_NNTB_ values of both the homologous series are comparable. A bent geometry is advantageous, whereas a more linear molecular shape more likely to stabilize the conventional N phase. Inducing the N_TB_ phase in CBOCO*n*SBA(CN) requires more supercooled conditions from the N phase upon cooling compared with inducing this phase in the more bent CBCOO*n*SBA(CN) [34,82]. Accordingly, the *T*_NNTB_ values of more linear CBOCO*n*SBA(CN) are comparable to those of more bent CBCOO*n*SBA(CN), in contrast to the trend observed for *T*_IN_ values between both series. Table 1 and Table 2 reveal that the *T*_NNTB_/*T*_IN_ values used to indicate the supercooling degree of the N_TB_ phase formation from *T*_IN_ [83] are lower for CBOCO*n*SBA(CN) (0.82–0.85) than those for CBCOO*n*SBA(CN) (0.88–0.95).

The *T*_IN_ and *T*_NNTB_ values of CBCOO*n*SBA(CN) decrease with decreasing *n* values from 10 to 4 and marginally increase at *n* = 2. This reduction could be ascribed to the enhanced effective molecular biaxiality or molecular bend for the shorter *n* and is partly supported by the Δ*S*_IN_/*R* trend, which decreases with descending *n*. The exception for *n* = 2 could be attributed to its high rigidity owing to its short spacers.

However, the trends of *T*_IN_ and *T*_NNTB_ values as a function of *n* for CBOCO*n*SBA(CN) differ from those for CBCOO*n*SBA(CN). Specifically, the *T*_IN_ and *T*_NNTB_ values of CBOCO*n*SBA(CN) are comparable or marginally increase with decreasing *n* and suddenly decrease at the shortest spacer (*n* = 2). These trends resemble those of the structurally similar cyanobiphenyl-based CBOCO*n*SCB [77] and ether-linked CBO*n*OCB [84]. The bond angle of Ar–O–C in the OCO-ester is similar to the ~118° bond angle in ether. Therefore, trends for *n* of OCO-ester-linked dimers are similar to those of ether-linked ones in terms of the molecular shape (bend). The *T*_IN_ and *T*_NNTB_ values of the OCO-ester-linked and ether-linked dimers with shorter spacers are expected to be compensated by molecular anisotropy and flexibility owing to the Ar–O–C ether bond. More anisotropy increases *T*_IN_, whereas more flexibility is likely to widen the conformations and distort the effective molecular bent shape of OCO-linked dimers compared to those of the more bent COO-linked dimers.

### 3.2. Photoinduced Phase Transitions

**Figure 9:**
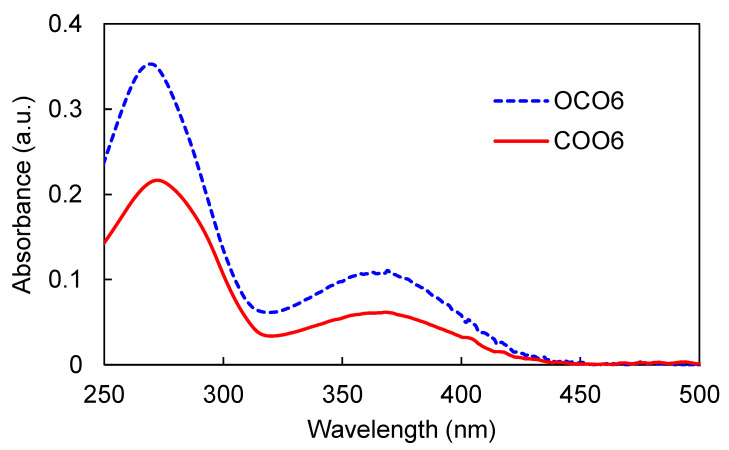
Non-normalized UV–visible spectra of CBCOO6SBA(CN) (red solid line) and CBOCO6SBA(CN) (blue dashed line) in THF solution.

The UV–visible spectra of *n* = 6 for both series before UV irradiation recorded in dilute THF solutions are shown in Figure 9. These spectra are similar, exhibiting two main absorption peaks. A main band was observed at approximately 370 nm, attributed to the π–π* transition of the benzylideneaniline moiety [34]. Because of the presence of the thioether linkage and cyano group, the absorption wavelengths from the π–π* transitions are red-shifted compared to around 310 nm of typical benzylideneanilines [85,86]. In addition, a peak at approximately 265 nm in their spectra may be ascribed to the σ–π* transition of the benzylideneaniline moiety [85,86] and the π–π* transition of the cyanobiphenyl moiety [87]. These results agree with those of previously reported thioether-linked *N*-benzylideneaniline-based LC dimers [34].

**Figure 10:**
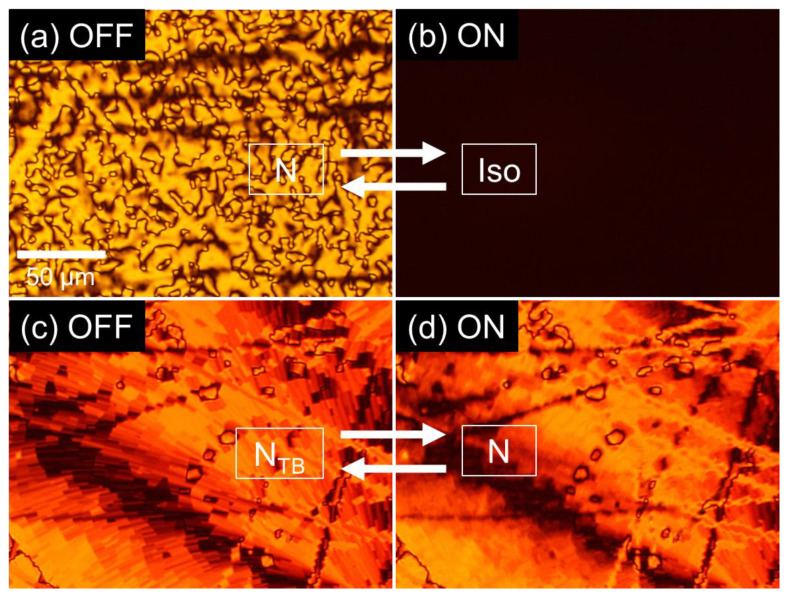
POM images under UV irradiation for CBCOO6SBA(CN). Turning UV (**a**) on and (**b**) off at 106.0 °C and turning UV (**c**) on and (**d**) off at 85.6 °C in a non-treated glass cell.

The photoinduced phase transitions of CBCOO*n*SBA(CN) and CBOCO*n*SBA(CN) were examined by irradiating UV light (365 nm and approximately 3 mW cm^−2^) to the N and N_TB_ phases of these dimers. This applied wavelength of the light source is appropriate as the UV–visible absorption is observed at approximately 370 nm for the π–π* transition of the benzylideneaniline moiety in both dimers. The optical textures of the LC phases of CBCOO6SBA(CN) when the UV light is turned on and off are shown in Figure 10. Upon UV irradiation, the birefringent N phase texture changed to the dark textures of the Iso phase, and the birefringent texture recovered after turning off the UV light, shown in Figure 10a,b, indicating a reversible photoinduced phase transition between the N and Iso phases. However, UV irradiation turned the blocky textures of the N_TB_ phase into the marble texture of the N phase, which then reversibly returned to the initial N_TB_ phase texture after the UV irradiation was turned off, as shown in Figure 10c,d. This behavior revealed a reversible photoinduced phase transition between the N_TB_ and N phases. These photoinduced phase transition behaviors are similar to those observed for azobenzene-based twist–bend nematogenic dimers [75,76]. To the best of our knowledge, this is the first report of photoinduced phase transitions in imine-based LC dimers. However, the temperature ranges for the transitions under UV light were strictly limited to 1 °C below the corresponding N or Iso phases. Similar azobenzene-based dimers exhibited photoinduced phase transitions within wider temperature ranges (~10 °C) using the same UV irradiation source. This difference could be ascribed to the energies of the isomerization process, as described in Section 1. The energy barrier of the *trans*-to-*cis* isomerization for the imine bond is higher than that of the azo bond [46,47,48], which may prevent the imine bond isomerization at lower temperatures and narrow the possible photoinduced phase-transition temperature ranges. Additionally, the *cis*-to-*trans* relaxation of the imine bond is remarkably faster owing to its lower energy barrier (~16 kcal mol^−1^) compared to that of azobenzene (23 kcal mol^−1^) [45]. We also recorded the UV-visible spectra of the samples in a THF solution subjected to UV irradiation for 1–15 min at room temperature. No changes were observed in the UV–visible spectra, indicating that the *N*-benzylideneaniline moieties exist in the *trans* form after UV irradiation, which agrees with the previously reported results [45,46]. One possibility is that the N–Iso and N_TB_–N phase transitions of the imine-based dimers upon UV–light irradiation might have been caused by the heat generated during the UV irradiation, considering their limited temperature windows. However, the texture changed reversibly soon after switching on and off the light. In addition, extending the UV irradiation time caused neither further changes in textures nor expansion of the area. Therefore, the phase transitions upon UV irradiation could have been driven by the photoisomerization of the imine bond. Owing to the limitations of the apparatus, we could not use incident light higher than approximately 3 mW cm^−2^. High-intensity light may expand the temperature range of photoinduced phase transitions and lead to the N_TB_–Iso phase transition, as observed for an azobenzene dimer [75].

## 4. Conclusions

We synthesized two homologous series of *N*-(4-cyanobenzylidene)aniline and cyanobiphenyl-based LC dimers with thioether and inverse ester linkages (COO and OCO, respectively). The dimers formed a monotropic N_TB_ phase below the temperature of the conventional N phase. Remarkably, the N_TB_ phases of certain CBCOO*n*SBA(CN) and CBOCO*n*SBA(CN) homologs were stably supercooled to ambient temperatures and vitrified. The LC phase-transition temperatures (*T*_IN_ and *T*_NNTB_) and associated entropy changes (Δ*S*_IN_) of the CBCOO*n*SBA(CN) homologs were lower than those of the CBOCO*n*SBA(CN) homologs at the same *n*, which could be attributed to the more bent molecular geometry of the CBCOO*n*SBA(CN). UV irradiation transformed the N_TB_ and N phases of the dimers into the N and Iso phases, respectively. These phases reversibly returned to their initial phases after turning off the UV light. This study successfully demonstrates the photoinduced phase transitions of *N*-benzylideneaniline-based LC dimers with the N_TB_ phases, which show a remarkable potential for photomobile LC materials.

## Figures and Tables

**Table 1:** Values of *T*_m_ upon heating, *T*_IN_, *T*_NNTB_, *T*_g_, and *T*_Cr_ upon cooling at a rate of 10 °C min^–1^ and their associated Δ*H* for CBCOO*n*SBA(CN).

*n*	*T*_m_ (°C)	Δ*H*		*T*_IN_ (°C)	Δ*H*	Δ*S*_IN_		*T*_NNTB_ (°C)	Δ*H*		*T*_g_ or *T*_Cr_ (°C)	Δ*H*	
		(kJ mol^–1^)			(kJ mol^–1^)				(kJ mol^–1^)			(kJ mol^–1^)	
2	170.11	82.93	I	95.0	0.27	0.05	N	50.91	0.06	N_TB_	24.63	-	G
4	124.70	68.26	I	84.9	0.12	0.06	N	59.24	0.24	N_TB_	13.7	-	G
6	124.37	80.67	I	102.0	0.38	0.12	N	82.01	0.8	N_TB_	16.54	-	G
8	90.32	50.3	I	115.6	0.93	0.29	N	91.16	-	N_TB_	19.95	-	G
10	108.84	79.99	I	115.7	1.43	0.44	N	90.26	-	N_TB_	60.75	24.97	Cr

**Table 2:** Values of *T*_m_ upon heating, *T*_IN_, *T*_NNTB_, *T*_g_, and *T*_Cr_ upon cooling at a rate of 10 °C min^–1^ and their associated Δ*H* for CBOCO*n*SBA(CN).

*n*	*T*_m_ (°C)	Δ*H*		*T*_IN_ (°C)	Δ*H*	Δ*S*_IN_		*T*_NNTB_ (°C)	Δ*H*		*T*_g_ or *T*_Cr_ (°C)	Δ*H*	
		(kJ mol^–1^)			(kJ mol^–1^)				(kJ mol^–1^)			(kJ mol^–1^)	
2	156.47	36.59	I	139.4	1.39	0.41	N	66	-	N_TB_^a^	104.3	27.1	Cr
4	142.1	43.66	I	161.7	1.50	0.42	N	88.5	-	N_TB_^a^	94.65	33.8	Cr
6	108.8	38.28	I	160.8	2.13	0.59	N	93.75	-	N_TB_	8.31	-	G
8	111.33	40.47	I	156.8	2.83	0.79	N	91.84	-	N_TB_	7.44	-	G
10	113.87	43.44	I	150.7	3.39	0.96	N	86.17	-	N_TB_	68.94	30.3	Cr

^a^ Determined by POM.

## Data Availability

Data are presented in the article and Supplementary Materials.

## Supplementary Materials

The following supporting information can be downloaded at: https://www.mdpi.com/article/10.3390/ma17133278/s1.
