# Supplementary material for: Photoinduced Phase Transitions of Imine-Based Liquid Crystal Dimers with Twist–Bend Nematic Phases
Source: Materials (Basel). 2024 Jul 3;17(13):3278. doi: 10.3390/ma17133278 (PMC11243583; doi:10.3390/ma17133278)
Supplement: Supplementary file 1 [file materials-17-03278-s001.zip › materials-3058788-supplementary.pdf]

Supporting information for

Photoinduced Phase Transitions of Imine-Based Liquid Crystal Dimers with Twist–Bend Nematic Phases

Yuki Arakawa * and Yuto Arai

Department of Applied Chemistry and Life Science, Graduate School of Engineering, Toyohashi University of Technology, 1-1 Hibarigaoka, Tempaku-cho, Toyohashi 441-8580, Aichi, Japan; arai.yuto.du@tut.jp
* Correspondence: arakawa@tut.jp

Synthesis

The typical synthetic procedures of n = 6 for both series are described below. Similar procedures were basically taken for all the homologues.

Synthesis of *CBCOOnSBA(CN)* series

CBCOOH

4-Bromobenzoic acid (2.21 g, 10.9 mmol), 4-(4,4,5,5-tetramethyl-1,3,2-dioxabororane-2-yl)benzonitrile (2.50 g, 10.9 mmol), Cs_2CO_3 (7.10 g, 21.8 mmol), and $Pd(PPh_3)_4$ (0.378 g, 0.327 mmol) were put in a two-necked round-bottomed flask purged with an argon gas. 1,4-Dioxane (25 mL) bubbled with an argon gas was added into the flask and the mixture was stirred at 100 ºC for 18h. The reaction mixture was poured into a beaker and acidified to pH = 2 with 2M HCl aq. The solid was collected by filtration and washed with a plenty amount of distilled water. The filtrated solid was dried in a vacuum oven and recrystallized in an arbitrarily mixed methanol/acetone solvent. ^{1}H NMR (500 MHz, DMSO-d_6) δ 8.04 (d, J = 8.5 Hz, Ar–*H*, 2H), 7.95 (d, J = 9.0 Hz, Ar–*H*, 2H), 7.92 (d, J = 8.5 Hz, Ar–*H*, 2H), 7.86 (d, J = 8.5 Hz, Ar–*H*, 2H) ppm. ^{13}C NMR (125 MHz, DMSO-d_6) δ 170.0, 143.5, 142.3, 133.0, 130.8, 130.1, 127.9, 127.4, 118.8, 110.9 ppm.

CBCOOnBr

CBCOO6Br

CBCOOH (560 mg, 2.50 mmol), 6-bromohexanol (458 mg, 2.53 mmol), 4-dimethylaminopyridine (DMAP) (31 mg, 0.254 mmol), and *N,N'*-dicyclohexylcarbodiimide (DCC) (786 mg, 3.81 mmol) were put in a double-necked flask purged with an argon gas. Then, dehydrated dichloromethane (8 mL) was added into the flask in an ice bath. The mixture was stirred at ambient temperature for 6h and filtrated off to remove the urea. After evaporating solvents and volatiles under reduced pressure, the residue was purified by column chromatography on a silica gel with an eluent of a dichloromethane/hexane (2/1, v/v) mixed solvent. ^{1}H NMR (500 MHz, $CDCl_3$) δ 8.15 (d, J = 8.5 Hz, Ar–*H*, 2H), 7.76 (d, J = 8.0 Hz, Ar–*H*, 2H), 7.72 (d, J = 8.5 Hz, Ar–*H*, 2H), 7.66 (d, J = 8.5 Hz, Ar–*H*, 2H), 4.36 (t, J = 6.5 Hz, Ar–(C=O)–O–CH_2, 2H), 3.43 (t, J = 6.8 Hz, Br–CH_2, 2H), 1.91 (tt, J = 7.1 and 6.8 Hz, Br–CH_2–CH_2, 2H), 1.82 (tt, J = 7.0 and 6.5 Hz, Ar–(C=O)–O–CH_2–CH_2, 2H), 1.58–1.47 (m, Ar–(C=O)–O–CH_2–CH_2–CH_2–CH_2, 4H) ppm. ^{13}C NMR (125 MHz, $CDCl_3$) δ 166.1, 144.4, 143.4, 132.7, 130.4, 130.3, 127.9, 127.2, 118.7, 65.1, 33.7, 32.6, 28.5, 27.8, 25.3 ppm.

CBCOO2Br

^{1}H NMR (500 MHz, $CDCl_3$) δ 8.18 (d, *J* = 8.5 Hz, Ar–*H*, 2H), 7.77 (d, *J* = 9.0 Hz, Ar–*H*, 2H), 7.72 (d, *J* = 8.0 Hz, Ar–*H*, 2H), 7.68 (d, *J* = 8.5 Hz, Ar–*H*, 2H), 4.67 (t, *J* = 6.0 Hz, Ar–(C=O)–O–CH_2, 2H), 3.68 (t, *J* = 6.3 Hz, Br–CH_2, 2H) ppm. ^{13}C NMR (125 MHz, $CDCl_3$) δ 165.6, 144.3, 143.8, 132.7, 130.5, 129.6, 127.9, 127.3, 118.6, 111.9, 64.4, 28.8 ppm.

CBCOO4Br

^{1}H NMR (500 MHz, $CDCl_3$) δ 8.14 (d, *J* = 8.5 Hz, Ar–*H*, 2H), 7.77 (d, *J* = 8.5 Hz, Ar–*H*, 2H), 7.72 (d, *J* = 8.5 Hz, Ar–*H*, 2H), 7.66 (d, *J* = 8.5 Hz, Ar–*H*, 2H), 4.40 (t, *J* = 6.5 Hz, Ar–(C=O)–O–CH_2, 2H), 3.50 (t, *J* = 6.5 Hz, Br–CH_2, 2H), 2.06 (tt, *J* = 6.6 and 6.5 Hz, Br–CH_2–CH_2, 2H), 1.98 (tt, *J* = 6.6 and 6.5 Hz, Ar–(C=O)–O–CH_2–CH_2, 2H) ppm. ^{13}C NMR (125 MHz, $CDCl_3$) δ 166.0, 144.4, 143.5, 132.7, 130.3, 130.2, 127.9, 127.3, 118.6, 111.9, 64.2, 33.0, 29.4, 27.4 ppm.

CBCOO8Br

^{1}H NMR (500 MHz, $CDCl_3$) δ 8.15 (d, *J* = 8.5 Hz, Ar–*H*, 2H), 7.76 (d, *J* = 8.5 Hz, Ar–*H*, 2H), 7.72 (d, *J* = 8.5 Hz, Ar–*H*, 2H), 7.66 (d, *J* = 8.5 Hz, Ar–*H*, 2H), 4.35 (t, *J* = 6.8 Hz, Ar–(C=O)–O–CH_2, 2H), 3.41 (t, *J* = 6.5 Hz, Br–CH_2, 2H), 1.87 (tt, *J* = 7.1 and 6.5 Hz, Br–CH_2–CH_2, 2H), 1.80 (tt, *J* = 7.1 and 6.8 Hz, Ar–(C=O)–O–CH_2–CH_2, 2H), 1.50–1.32 (m, Ar–(C=O)–O–CH_2–CH_2–CH_2–CH_2–CH_2–CH_2, 8H) ppm. ^{13}C NMR (125 MHz, $CDCl_3$) δ 166.2, 144.5, 143.3, 132.7, 130.5, 130.3, 127.9, 127.2, 118.7, 111.8, 65.3, 33.9, 32.7, 29.0, 28.7, 28.6, 28.0, 25.9 ppm.

CBCOO10Br

^{1}H NMR (500 MHz, $CDCl_3$) δ 8.15 (d, *J* = 8.0 Hz, Ar–*H*, 2H), 7.76 (d, *J* = 8.5 Hz, Ar–*H*, 2H), 7.72 (d, *J* = 8.0 Hz, Ar–*H*, 2H), 7.66 (d, *J* = 8.5 Hz, Ar–*H*, 2H), 4.35 (t, *J* = 6.8 Hz, Ar–(C=O)–O–CH_2, 2H), 3.41 (t, *J* = 6.8 Hz, Br–CH_2, 2H), 1.85 (tt, *J* = 7.3 and 6.8 Hz, Br–CH_2–CH_2, 2H), 1.79 (tt, *J* = 7.1 and 6.8 Hz, Ar–(C=O)–O–CH_2–CH_2, 2H), 1.48–1.25 (m, Ar–(C=O)–O–CH_2–CH_2–CH_2–CH_2–CH_2–CH_2–CH_2–CH_2–CH_2, 12H) ppm. ^{13}C NMR (125 MHz, $CDCl_3$) δ 166.2, 144.5, 143.3, 132.7, 130.6, 130.3, 127.9, 127.2, 118.7, 111.8, 65.3, 34.0, 32.8, 29.4, 29.3, 29.2, 28.70, 28.68, 28.1, 26.0 ppm.

CBCOOnSAP

CBCOO6SAP

A mixture of CBCOO6Br (579 mg, 1.5 mmol), 4-aminobenzenethiol (0.188 g, 1.50

mmol), K_2CO_3 (0.426 g, 3.08 mmol), and acetonitrile (6 mL) was stirred in a flask at ambient temperature for 19 h. The reaction mixture was extracted with dichloromethane and washed with water and brine. The organic phase was dried over $MgSO_4$ and the volatiles were removed under reduced pressure. The residue was purified by column chromatography on a silica gel with an eluent of a dichloromethane/ethyl acetate (30/1, v/v) mixed solvent. ^{1}H NMR (500 MHz, $CDCl_3$) δ 8.14 (d, *J* = 8.5 Hz, Ar–*H*, 2H), 7.76 (d, *J* = 8.0 Hz, Ar–*H*, 2H), 7.72 (d, *J* = 8.5 Hz, Ar–*H*, 2H), 7.65 (d, *J* = 8.5 Hz, Ar–*H*, 2H), 7.23 (d, *J* = 9.0 Hz, Ar–*H*, 2H), 6.61 (d, *J* = 8.5 Hz, Ar–*H*, 2H), 4.33 (t, *J* = 6.8 Hz, Ar–(C=O)–O–CH_2, 2H), 3.70 (s, Ar–NH_2, 2H), 2.78 (t, *J* = 7.3 Hz, Ar–S–CH_2, 2H), 1.77 (tt, *J* = 6.9 and 6.8 Hz, Ar–(C=O)–O–CH_2–CH_2, 2H), 1.61 (tt, *J* = 7.1 and 7.3 Hz, Ar–S–CH_2–CH_2, 2H), 1.51–1.41 (m, Ar–(C=O)–O–CH_2–CH_2–CH_2–CH_2, 4H) ppm. ^{13}C NMR (125 MHz, $CDCl_3$) δ 166.1, 145.7, 144.5, 143.3, 133.8, 132.7, 130.5, 130.3, 127.9, 127.2, 123.6, 118.7, 115.5, 111.8, 65.2, 36.3, 29.2, 28.6, 28.3, 25.6 ppm.

CBCOO2SAP

^{1}H NMR (500 MHz, $CDCl_3$) δ 8.08 (d, *J* = 8.5 Hz, Ar–*H*, 2H), 7.76 (d, *J* = 8.5 Hz, Ar–*H*, 2H),7.71 (d, *J* = 8.0 Hz, Ar–*H*, 2H), 7.64 (d, *J* = 8.5 Hz, Ar–*H*, 2H), 7.32 (d, *J* = 8.5 Hz, Ar–*H*, 2H), 6.63 (d, *J* = 8.5 Hz, Ar–*H*, 2H), 4.46 (t, *J* = 6.8 Hz, Ar–(C=O)–O–CH_2, 2H), 3.75 (s, Ar–NH_2, 2H), 3.12 (t, *J* = 6.8 Hz, Ar–S–CH_2, 2H) ppm. ^{13}C NMR (125 MHz, $CDCl_3$) δ 165.9, 146.5, 144.4, 143.4, 134.8, 132.7, 130.4, 130.1, 127.9, 127.2, 121.8, 118.7, 115.6, 111.8, 63.8, 35.0 ppm.

CBCOO4SAP

^{1}H NMR (500 MHz, $CDCl_3$) δ 8.11 (d, *J* = 8.0 Hz, Ar–*H*, 2H), 7.76 (d, *J* = 8.0 Hz, Ar–*H*, 2H), 7.72 (d, *J* = 8.0 Hz, Ar–*H*, 2H), 7.66 (d, *J* = 8.0 Hz, Ar–*H*, 2H), 7.25 (d, *J* = 8.5 Hz, Ar–*H*, 2H), 6.61 (d, *J* = 8.5 Hz, Ar–*H*, 2H), 4.34 (t, *J* = 6.5 Hz, Ar–(C=O)–O–CH_2, 2H), 3.71 (s, Ar–NH_2, 2H), 2.85 (t, *J* = 7.3 Hz, Ar–S–CH_2, 2H), 1.91 (tt, *J* = 7.0 and 6.5 Hz, Ar–(C=O)–O–CH_2–CH_2, 2H), 1.74 (tt, *J* = 7.4and 7.3 Hz, Ar–S–CH_2–CH_2, 2H) ppm. ^{13}C NMR (125 MHz, $CDCl_3$) δ 166.1, 146.0, 144.4, 143.3, 134.1, 132.7, 130.4, 130.3, 127.9, 127.2, 123.0, 118.7, 115.6, 111.8, 64.7, 36.0, 27.6, 25.8 ppm.

CBCOO8SAP

^{1}H NMR (500 MHz, $CDCl_3$) δ 8.14 (d, *J* = 8.5 Hz, Ar–*H*, 2H), 7.76 (d, *J* = 8.5 Hz, Ar–*H*, 2H), 7.71 (d, *J* = 8.5 Hz, Ar–*H*, 2H), 7.66 (d, *J* = 8.5 Hz, Ar–*H*, 2H), 7.22 (d, *J* = 8.5 Hz, Ar–*H*, 2H), 6.61 (d, *J* = 8.5 Hz, Ar–*H*, 2H), 4.34 (t, *J* = 6.5 Hz, Ar–(C=O)–O–CH_2,

2H), 3.69 (s, Ar–N*H*$_2$, 2H), 2.76 (t, *J* = 7.5 Hz, Ar–S–C*H*$_2$, 2H), 1.78 (tt, *J* = 7.1 and 6.5 Hz, Ar–(C=O)–O–CH$_2$–C*H*$_2$, 2H), 1.57 (tt, *J* = 7.4 and 7.5 Hz, Ar–S–CH$_2$–C*H*$_2$, 2H), 1.47–1.25 (m, Ar–(C=O)–O–CH$_2$–CH$_2$–C*H*$_2$–C*H*$_2$–C*H*$_2$–C*H*$_2$, 8H) ppm. ^{13}C NMR (125 MHz, CDCl$_3$) δ 166.2, 145.7, 144.5, 143.3, 133.7, 132.7, 130.5, 130.3, 127.9, 127.2, 123.7, 118.7, 115.5, 111.8, 65.3, 36.3, 29.3, 29.1, 29.0, 28.7, 28.5, 25.9 ppm.

CBCOO10SAP

^{1}H NMR (500 MHz, CDCl$_3$) δ 8.14 (d, *J* = 8.0 Hz, Ar–*H*, 2H), 7.74 (d, *J* = 7.5 Hz, Ar–*H*, 2H), 7.71 (d, *J* = 8.0 Hz, Ar–*H*, 2H), 7.65 (d, *J* = 8.0 Hz, Ar–*H*, 2H), 7.22 (d, *J* = 8.0 Hz, Ar–*H*, 2H), 6.61 (d, *J* = 8.0 Hz, Ar–*H*, 2H), 4.34 (t, *J* = 6.5 Hz, Ar–(C=O)–O–C*H*$_2$, 2H), 3.72 (s, Ar–N*H*$_2$, 2H), 2.75 (t, *J* = 7.3 Hz, Ar–S–C*H*$_2$, 2H), 1.78 (tt, *J* = 7.0 and 6.5 Hz, Ar–(C=O)–O–CH$_2$–C*H*$_2$, 2H), 1.55 (tt, *J* = 7.1and 7.3 Hz, Ar–S–CH$_2$–C*H*$_2$, 2H), 1.44 (tt, *J* = 7.3 Hz, Ar–(C=O)–O–CH$_2$–CH$_2$–C*H*$_2$, 2H), 1.40–1.15 (m, Ar–(C=O)–O–CH$_2$–CH$_2$–CH$_2$–C*H*$_2$–C*H*$_2$–C*H*$_2$–C*H*$_2$–C*H*$_2$, 10H) ppm. ^{13}C NMR (125 MHz, CDCl$_3$) δ 166.1, 145.7, 144.4, 143.2, 133.6, 132.6, 130.5, 130.2, 127.8, 127.1, 123.6, 118.6, 115.4, 111.6, 65.3, 36.3, 29.30, 29.28, 29.25, 29.1, 29.0, 28.59, 28.53, 25.9 ppm.

CBCOOnSBA(CN)

CBCOO6SBA(CN)

A mixture of CBCOO6SAP (151 mg, 0.35 mmol), 4-formylbenzonitrile(55 mg, 0.42 mmol), *p*-toluenesulfonic acid (3.65 mg, 19.2 μmol), and ethanol (5 mL) was stirred at 70ºC for 4h. After cooled to ambient temperature, the solid was collected via filtration, washed with ethanol, and recrystallized in a chloroform/ethanol mixed solvent, affording the target compound. ^{1}H NMR (500 MHz, CDCl$_3$) δ 8.51 (s, Ar–N=CH–Ar, 1H), 8.14 (d, *J* = 8.5 Hz, Ar–H, 2H), 7.99 (d, *J* = 8.0 Hz, Ar–H, 2H), 7.76 (d, *J* = 9.0 Hz, Ar–H, 4H), 7.71 (d, *J* = 8.5 Hz, Ar–H, 2H), 7.65 (d, *J* = 8.5 Hz, Ar–H, 2H), 7.36 (d, *J* = 8.0 Hz, Ar–H, 2H), 7.19 (d, *J* = 8.0 Hz, Ar–H, 2H), 4.35 (t, *J* = 6.8 Hz, Ar–(C=O)–O–C*H*$_2$, 2H), 2.97 (t, *J* = 7.3 Hz, Ar–S–C*H*$_2$, 2H), 1.80 (tt, *J* = 7.0 and 6.8 Hz, Ar–(C=O)–O–CH$_2$–C*H*$_2$, 2H), 1.72 (tt, *J* = 7.3 and 7.3 Hz, Ar–S–CH$_2$–C*H*$_2$, 2H), 1.56–1.46 (m, Ar–(C=O)–O–CH$_2$–CH$_2$–C*H*$_2$–C*H*$_2$, 4H) ppm. ^{13}C NMR (125 MHz, CDCl$_3$) δ 166.1, 157.2, 148.6, 144.4, 143.4, 139.9, 135.8, 132.7, 132.5, 130.5, 130.3, 129.7, 129.0, 127.9, 127.2, 121.6, 118.6, 118.4, 114.4, 111.8, 65.1, 33.7, 28.9, 28.6, 28.4, 25.6 ppm.

CBCOO2SBA(CN)

^{1}H NMR (500 MHz, $CDCl_3$) δ 8.47 (s, Ar–N=C*H*, 1H), 8.09 (d, *J* = 8.5 Hz, Ar–*H*, 2H), 7.99 (d, *J* = 8.5 Hz, Ar–*H*, 2H), 7.76 (d, *J* = 8.0 Hz, Ar–*H*, 2H), 7.74 (d, *J* = 8.0 Hz, Ar–*H*, 2H), 7.70 (d, *J* = 8.0 Hz, Ar–*H*, 2H), 7.64 (d, *J* = 8.0 Hz, Ar–*H*, 2H), 7.49 (d, *J* = 8.5 Hz, Ar–*H*, 2H), 7.21 (d, *J* = 8.5 Hz, Ar–*H*, 2H), 4.55 (t, *J* = 6.8 Hz, Ar–(C=O)–O–CH_2, 2H), 3.33 (t, *J* = 6.8 Hz, Ar–S–CH_2, 2H) ppm. ^{13}C NMR (125 MHz, $CDCl_3$) δ 165.8, 157.7, 149.5, 144.3, 143.6, 139.8, 133.7, 132.7, 132.5, 131.0, 130.4, 129.8, 129.1, 127.9, 127.2, 121.8, 118.6, 118.4, 114.5, 111.9, 63.6, 32.8 ppm.

CBCOO4SBA(CN)

^{1}H NMR (500 MHz, $CDCl_3$) δ 8.49 (s, Ar–N=C*H*, 1H), 8.11 (d, *J* = 8.0 Hz, Ar–*H*, 2H), 7.98 (d, *J* = 8.0 Hz, Ar–*H*, 2H), 7.75 (d, *J* = 8.0 Hz, Ar–*H*, 2H), 7.74 (d, *J* = 8.0 Hz, Ar–*H*, 2H), 7.70 (d, *J* = 8.0 Hz, Ar–*H*, 2H), 7.65 (d, *J* = 8.0 Hz, Ar–*H*, 2H), 7.39 (d, *J* = 8.5 Hz, Ar–*H*, 2H), 7.19 (d, *J* = 8.5 Hz, Ar–*H*, 2H), 4.38 (t, *J* = 6.3 Hz, Ar–(C=O)–O–CH_2, 2H), 3.04 (t, *J* = 7.3 Hz, Ar–S–CH_2, 2H), 1.97 (tt, *J* = 7.0 and 6.3 Hz, Ar–(C=O)–O–CH_2–CH_2, 2H), 1.85 (tt, *J* = 7.4 and 7.3 Hz, Ar–S–CH_2, 2H) ppm. ^{13}C NMR (125 MHz, $CDCl_3$) δ 166.0, 157.3, 148.8, 144.4, 143.4, 139.8, 135.1, 132.7, 132.5, 130.30, 130.26, 130.1, 129.0, 127.9, 127.2, 121.7, 118.6, 118.4, 114.4, 111.8, 64.5, 33.5, 27.7, 25.6 ppm.

CBCOO8SBA(CN)

^{1}H NMR (500 MHz, $CDCl_3$) δ 8.51 (s, Ar–N=CH–Ar, 1H), 8.14 (d, *J* = 8.5 Hz, Ar–H, 2H), 8.00 (d, *J* = 8.5 Hz, Ar–H, 2H), 7.76 (d, *J* = 8.0 Hz, Ar–H, 4H), 7.71 (d, *J* = 8.5 Hz, Ar–H, 2H), 7.65 (d, *J* = 8.0 Hz, Ar–H, 2H), 7.35 (d, *J* = 8.5 Hz, Ar–H, 2H), 7.20 (d, *J* = 8.0 Hz, Ar–H, 2H), 4.35 (t, *J* = 6.8 Hz, Ar–(C=O)–O–CH_2, 2H), 2.95 (t, *J* = 7.3 Hz, Ar–S–CH_2, 2H), 1.79 (tt, *J* = 7.0 and 6.8 Hz, Ar–(C=O)–O–CH_2–CH_2, 2H), 1.68 (tt, *J* = 7.5 and 7.3 Hz, Ar–S–CH_2–CH_2, 2H), 1.46 (tt, *J* = 7.1 Hz, Ar–(C=O)–O–CH_2–CH_2–CH_2 and Ar–S–CH_2–CH_2–CH_2, 4H), 1.41–1.32 (m, Ar–(C=O)–O–CH_2–CH_2–CH_2–CH_2–CH_2, 4H) ppm. ^{13}C NMR (125 MHz, $CDCl_3$) δ 166.3, 157.2, 148.6, 144.5, 143.4, 140.0, 136.1, 132.8, 132.6, 130.6, 130.4, 129.7, 129.1, 128.0, 127.3, 121.7, 118.8, 118.5, 111.9, 65.4, 33.9, 29.2, 29.12, 29.10, 28.78, 28.76, 26.0 ppm.

CBCOO10SBA(CN)

^{1}H NMR (500 MHz, $CDCl_3$) δ 8.51 (s, Ar–N=CH–Ar, 1H), 8.14 (d, *J* = 8.5 Hz, Ar–H, 2H), 8.00 (d, *J* = 8.5 Hz, Ar–H, 2H), 7.76 (d, *J* = 8.5 Hz, Ar–H, 4H), 7.71 (d, *J* = 8.5 Hz, Ar–H, 2H), 7.66 (d, *J* = 8.5 Hz, Ar–H, 2H), 7.35 (d, *J* = 8.5 Hz, Ar–H, 2H),

7.20 (d, *J* = 8.5 Hz, Ar–H, 2H), 4.35 (t, *J* = 6.8 Hz, Ar–(C=O)–O–C*H*$_2$, 2H), 2.94 (t, *J* = 7.5 Hz, Ar–S–C*H*$_2$, 2H), 1.79 (tt, *J* = 7.0 and 6.8 Hz, Ar–(C=O)–O–CH$_2$–C*H*$_2$, 2H), 1.66 (tt, *J* = 7.5 and 7.5 Hz, Ar–S–CH$_2$–C*H*$_2$, 2H), 1.48–1.41 (m, Ar–(C=O)–O–CH$_2$–CH$_2$–C*H*$_2$ and Ar–S–CH$_2$–CH$_2$–C*H*$_2$, 4H), 1.38–1.23 (m, Ar–(C=O)–O–CH$_2$–CH$_2$–CH$_2$–C*H*$_2$–C*H*$_2$–C*H*$_2$–C*H*$_2$, 8H) ppm. ^{13}C NMR (125 MHz, CDCl$_3$) δ 166.2, 157.1, 148.5, 144.4, 139.9, 136.0, 132.7, 132.5, 130.5, 130.3, 129.5, 129.0, 127.9, 127.2, 121.6, 111.8, 65.3, 33.7, 29.4, 29.2, 29.1, 29.0, 28.8, 28.7, 26.0 ppm.

Synthesis of CBOCO*n*SBA(CN) series

CBOCOnBr

CBOCO6Br

CBOH (594 mg, 3.04 mmol), 7-bromoheptanoic acid (625 mg, 2.99 mmol), 4-dimethylaminopyridine (DMAP) (36 mg, 0.295 mmol), and *N,N'*-dicyclohexylcarbodiimide (DCC) (0.932 g, 4.52 mmol) were put in a double-necked flask purged with an argon gas. Then, dehydrated dichloromethane (8 mL) was added into the flask in an ice bath. The mixture was stirred at ambient temperature for one day and filtrated off to remove the urea. After evaporating volatiles under reduced pressure, the residue was purified by column chromatography on a silica gel with an eluent of a dichloromethane/hexane (3/1, v/v) mixed solvent. ^{1}H NMR (400 MHz, CDCl$_3$) δ 7.73 (d, *J* = 8.4 Hz, Ar–*H*, 2H), 7.66 (d, *J* = 8.8 Hz, Ar–*H*, 2H), 7.59 (d, *J* = 8.8 Hz, Ar–*H*, 2H), 7.20 (d, *J* = 8.4 Hz, Ar–*H*, 2H), 3.43 (t, *J* = 6.6 Hz, Br–C*H*$_2$, 2H), 2.61 (t, *J* = 7.4 Hz, Ar–O–(C=O)–C*H*$_2$, 2H), 1.91 (tt, *J* = 7.0 and 7.4 Hz, Br–CH$_2$–C*H*$_2$, 2H), 1.80 (tt, *J* = 7.4 and 6.6 Hz, Ar–O–(C=O)–CH$_2$–C*H*$_2$, 2H), 1.55–1.43 (m, Ar–O–(C=O)–CH$_2$–CH$_2$–C*H*$_2$–C*H*$_2$, 4H) ppm. ^{13}C NMR (100 MHz, CDCl$_3$) δ 172.0, 151.1, 144.7, 136.8, 132.6, 128.3, 127.6, 122.3, 118.8, 111.0, 34.9, 34.2, 32.4, 28.1, 27.7, 24.7 ppm.

CBOCO2Br

^{1}H NMR (500 MHz, CDCl$_3$) δ 7.73 (d, *J* = 8.5 Hz, Ar–*H*, 2H), 7.66 (d, *J* = 8.0 Hz, Ar–*H*, 2H), 7.61 (d, *J* = 8.5 Hz, Ar–*H*, 2H), 7.24 (d, *J* = 9.0 Hz, Ar–*H*, 2H), 3.71 (t, *J* = 6.8 Hz, Br–C*H*$_2$, 2H), 3.22 (t, *J* = 6.8 Hz, Ar–O–(C=O)–C*H*$_2$,2H) ppm. ^{13}C NMR (125 MHz, CDCl$_3$) δ 169.0, 150.8, 144.6, 137.2, 132.7, 128.4, 127.7, 122.2, 118.8, 111.1,

37.8, 25.5 ppm.

CBOCO4Br

^{1}H NMR (400 MHz, $CDCl_3$) δ 7.72 (d, *J* = 8.4 Hz, Ar–*H*, 2H), 7.65 (d, *J* = 8.4 Hz, Ar–*H*, 2H), 7.59 (d, *J* = 8.8 Hz, Ar–*H*, 2H), 7.21 (d, *J* = 8.8 Hz, Ar–*H*, 2H), 3.47 (t, *J* = 6.4 Hz, Br–CH_2, 2H), 2.64 (t, *J* = 7.0 Hz, Ar–O–(C=O)–CH_2, 2H), 2.02 (tt, *J* = 6.4 and 6.6 Hz, Br–CH_2–CH_2, 2H), 1.94 (tt, *J* = 7.0 and 6.9 Hz, Ar–O–(C=O)–CH_2–CH_2, 2H) ppm. ^{13}C NMR (100 MHz, $CDCl_3$) δ 171.5, 151.0, 144.6, 136.8, 132.6, 128.3, 127.6, 122.2, 118.8, 111.0, 33.3, 32.9, 31.8, 23.4 ppm.

CBOCO8Br

^{1}H NMR (400 MHz, $CDCl_3$) δ 7.72 (d, *J* = 8.8 Hz, Ar–*H*, 2H), 7.65 (d, *J* = 6.8 Hz, Ar–*H*, 2H), 7.59 (d, *J* = 8.8 Hz, Ar–*H*, 2H), 7.20 (d, *J* = 8.8 Hz, Ar–*H*, 2H), 3.42 (t, *J* = 7.0 Hz, Br–CH_2, 2H), 2.59 (t, *J* = 7.4 Hz, Ar–O–(C=O)–CH_2, 2H), 1.87 (tt, *J* = 7.0 and 7.1 Hz, Br–CH_2–CH_2, 2H), 1.78 (tt, *J* = 7.4 and 7.4 Hz, Ar–O–(C=O)–CH_2–CH_2, 2H), 1.49–1.26 (m, Br–CH_2–CH_2–CH_2–CH_2–CH_2–CH_2, 8H) ppm. ^{13}C NMR (100 MHz, $CDCl_3$) δ 172.1, 151.2, 144.7, 136.7, 132.6, 128.3, 127.6, 122.3, 118.8, 111.0, 34.3, 33.9, 32.7, 29.0, 28.9, 28.5, 28.0, 24.8 ppm.

CBOCO10Br

^{1}H NMR (500 MHz, $CDCl_3$) δ 7.73 (d, *J* = 8.5 Hz, Ar–*H*, 2H), 7.66 (d, *J* = 8.5 Hz, Ar–*H*, 2H), 7.59 (d, *J* = 8.5 Hz, Ar–*H*, 2H), 7.20 (d, *J* = 8.5 Hz, Ar–*H*, 2H), 3.41 (t, *J* = 7.0 Hz, Br–CH_2, 2H), 2.59 (t, *J* = 7.2 Hz, Ar–O–(C=O)–CH_2, 2H), 1.86 (tt, *J* = 7.0 and 7.1 Hz, Br–CH_2–CH_2), 1.77 (tt, *J* = 7.2 and 7.5 Hz, Ar–O–(C=O)–CH_2–CH_2, 2H), 1.28–1.47 (m, Br–CH_2–CH_2–CH_2–CH_2–CH_2–CH_2–CH_2–CH_2, 12H) ppm. ^{13}C NMR (125 MHz, $CDCl_3$) δ 172.2, 151.2, 144.8, 136.7, 132.6, 128.3, 127.6, 122.3, 118.8, 111.0, 34.3, 34.0, 32.8, 29.30, 29.28, 29.15, 29.0, 28.7, 28.1, 24.9 ppm.

CBOCOnSAP

CBOCO6SAP

A mixture of CBOCO6Br (251 mg, 0.65 mmol), 4-aminobenzenethiol (89 mg, 0.711 mmol), K_2CO_3(135 mg, 0.977 mmol), and acetonitrile (3.5 mL) was stirred in a flask at ambient temperature for 20h. The reaction mixture was extracted with dichloromethane and washed with water and brine. The organic phase was dried over $MgSO_4$ and the volatiles were removed under reduced pressure. The residue

was purified by column chromatography on a silica gel with an eluent of a dichloromethane/ethyl acetate (30/1, v/v) mixed solvent. ^{1}H NMR (500 MHz, $CDCl_3$) δ 7.73 (d, *J* = 8.0 Hz, Ar–*H*, 2H), 7.66 (d, *J* = 8.0 Hz, Ar–*H*, 2H), 7.59 (d, *J* = 8.5 Hz, Ar–*H*, 2H), 7.24 (d, *J* = 8.0 Hz, Ar–*H*, 2H), 7.19 (d, *J* = 8.0 Hz, Ar–*H*, 2H), 6.62 (d, *J* = 8.5 Hz, Ar–*H*, 2H), 3.70 (s, Ar–NH_2, 2H), 2.78 (t, *J* = 7.3 Hz, Ar–S–CH_2, 2H), 2.58 (t, *J* = 7.5 Hz, Ar–O–(C=O)–CH_2, 2H), 1.76 (tt, *J* = 7.4 and 7.3 Hz, Ar–O–(C=O)–CH_2–CH_2, 2H), 1.60 (tt, *J* = 7.3 and 7.5 Hz, Ar–S–CH_2–CH_2, 2H), 1.50–1.39 (m, Ar–O–(C=O)–CH_2–CH_2–CH_2–CH_2, 4H) ppm. ^{13}C NMR (100 MHz, $CDCl_3$) δ 172.1, 151.1, 145.8, 144.7, 136.7, 133.8, 132.6, 128.3, 127.6, 123.5, 122.3, 118.8, 115.5, 110.9, 36.2, 34.2, 29.1, 28.6, 28.2, 24.7 ppm.

CBOCO2SAP

^{1}H NMR (400 MHz, $CDCl_3$) δ 7.72 (d, *J* = 8.4 Hz, Ar–*H*, 2H), 7.65 (d, *J* = 8.4 Hz, Ar–*H*, 2H), 7.58 (d, *J* = 9.2 Hz, Ar–*H*, 2H), 7.32 (d, *J* = 8.4 Hz, Ar–*H*, 2H), 7.20 (d, *J* = 8.8 Hz, Ar–*H*, 2H), 6.65 (d, *J* = 8.4 Hz, Ar–*H*, 2H), 3.77 (s, Ar–NH_2, 2H), 3.12 (t, *J* = 7.0 Hz, Ar–S–CH_2, 2H), 2.83 (t, *J* = 7.4 Hz, Ar–O–(C=O)–CH_2, 2H) ppm. ^{13}C NMR (100 MHz, $CDCl_3$) δ 170.4, 151.1, 146.7, 144.7, 136.9, 135.2, 133.9, 132.6, 128.3, 127.7, 122.3, 121.5, 115.6, 111.0, 34.8, 31.7 ppm.

CBOCO4SAP

^{1}H NMR (500 MHz, $CDCl_3$) δ 7.73 (d, *J* = 8.0 Hz, Ar–*H*, 2H), 7.66 (d, *J* = 8.0 Hz, Ar–*H*, 2H), 7.59 (d, *J* = 8.5 Hz, Ar–*H*, 2H), 7.25 (d, *J* = 6.5 Hz, Ar–*H*, 2H), 7.16 (d, *J* = 8.5 Hz, Ar–*H*, 2H), 6.63 (d, *J* = 8.5 Hz, Ar–*H*, 2H), 3.71 (s, Ar–NH_2, 2H), 2.83 (t, *J* = 7.3 Hz, Ar–S–CH_2, 2H), 2.59 (t, *J* = 7.5 Hz, Ar–O–(C=O)–CH_2, 2H), 1.89 (tt, *J* = 7.6 and 7.5 Hz, Ar–O–(C=O)–CH_2–CH_2, 2H), 1.70 (tt, *J* = 7.5 and 7.3 Hz, Ar–S–CH_2–CH_2, 2H) ppm. ^{13}C NMR (100 MHz, $CDCl_3$) δ 171.8, 151.1, 145.9, 144.8, 136.8, 134.0, 132.6, 128.3, 127.6, 123.1, 122.3, 115.6, 115.3, 111.0, 36.0, 33.9, 28.7, 23.8 ppm.

CBOCO8SAP

^{1}H NMR (400 MHz, $CDCl_3$) δ 7.72 (d, *J* = 8.4 Hz, Ar–*H*, 2H), 7.65 (d, *J* = 8.4 Hz, Ar–*H*, 2H), 7.59 (d, *J* = 8.4 Hz, Ar–*H*, 2H), 7.23 (d, *J* = 8.4 Hz, Ar–*H*, 2H), 7.20 (d, *J* = 8.8 Hz, Ar–*H*, 2H), 6.62 (d, *J* = 8.8 Hz, Ar–*H*, 2H), 3.72 (s, Ar–NH_2, 2H), 2.77 (t, *J* = 7.4 Hz, Ar–S–CH_2, 2H), 2.58 (t, *J* = 7.6 Hz, Ar–O–(C=O)–CH_2, 2H), 1.76 (tt, *J* = 7.4 and 7.5 Hz, Ar– O–(C=O)–CH_2–CH_2, 2H), 1.57 (tt, *J* = 7.6 and 7.3 Hz, Ar–S–CH_2–CH_2, 2H), 1.43–1.26 (m, Ar–S–CH_2–CH_2–CH_2–CH_2–CH_2–CH_2, 8H) ppm. ^{13}C NMR (100 MHz, $CDCl_3$) δ 172.2, 151.2, 145.7, 144.8, 136.7, 133.7, 132.6, 128.3, 127.6, 123.7,

122.3, 118.8, 115.5, 111.0, 36.3, 34.3, 29.3, 29.1, 29.0, 28.9, 28.5, 24.8 ppm.

CBOCO10SAP

^{1}H NMR (400 MHz, $CDCl_3$) δ 7.72 (d, *J* = 8.8 Hz, Ar–*H*, 2H), 7.66 (d, *J* = 8.8 Hz, Ar–*H*, 2H), 7.59 (d, *J* = 8.8 Hz, Ar–*H*, 2H), 7.23 (d, *J* = 8.8 Hz, Ar–*H*, 2H), 7.20 (d, *J* = 8.8 Hz, Ar–*H*, 2H), 6.62 (d, *J* = 8.4 Hz, Ar–*H*, 2H), 3.70 (s, Ar–NH_2, 2H), 2.76 (t, *J* = 7.4 Hz, Ar–S–CH_2, 2H), 2.58 (t, *J* = 7.4 Hz, Ar–O–(C=O)–CH_2, 2H), 1.77 (tt, *J* = 7.4 and 7.5 Hz, Ar–O–(C=O)–CH_2–CH_2, 2H), 1.56 (tt, *J* = 7.4 and 7.4 Hz, S–CH_2–CH_2, 2H), 1.45–1.19 (m, Ar–O–(C=O)–CH_2–CH_2–CH_2–CH_2–CH_2–CH_2–CH_2–CH_2, 12H) ppm. ^{13}C NMR (100 MHz, $CDCl_3$) δ 172.2, 151.2, 145.6, 144.8, 136.7, 133.7, 132.6, 128.3, 127.7, 123.8, 122.3, 118.9, 115.5, 111.0, 36.4, 34.4, 29.4, 29.3×2, 29.2, 29.11, 29.05, 28.6, 24.9 ppm.

CBOCOnSBA(CN)

CBOCO6SBA(CN)

A mixture of CBOCO6SAP (129 mg, 0.30 mmol), 4-formylbenzonitrile (47 mg, 0.358 mmol), *p*-toluenesulfonic acid (1.96 mg, 10 μmol), and ethanol (5 mL) was stirred at 70ºC for 21h. After cooled to ambient temperature, the solid was collected via filtration, washed with ethanol, and recrystallized in a chloroform/ethanol mixed solvent, affording the target compound. ^{1}H NMR (500 MHz, $CDCl_3$) δ 8.51 (s, Ar–N=CH–Ar, 1H), 7.99 (d, *J* = 8.0 Hz, Ar–H, 2H), 7.76 (d, *J* = 8.5 Hz, Ar–H, 2H), 7.72 (d, *J* = 8.5 Hz, Ar–H, 2H), 7.65 (d, *J* = 8.5 Hz, Ar–H, 2H), 7.58 (d, *J* = 8.5 Hz, Ar–H, 2H), 7.37 (d, *J* = 8.5 Hz, Ar–H, 2H), 7.20 (d, *J* = 8.0 Hz, Ar–H, 2H), 7.19 (d, *J* = 9.0 Hz, Ar–H, 2H), 2.97 (t, *J* = 7.5 Hz, Ar–S–CH_2, 2H), 2.60 (t, *J* = 7.8 Hz, Ar–O–(C=O)–CH_2, 2H), 1.78 (tt, *J* = 7.5 and 7.8 Hz, Ar–O–(C=O)–CH_2–CH_2, 2H), 1.68 (tt, *J* = 7.4 and 7.5 Hz, Ar–S–CH_2–CH_2, 2H), 1.53 (tt, *J* = 7.7 and 7.4 Hz, Ar–S–CH_2–CH_2–CH_2, 2H), 1.46 (tt, *J* = 7.6 and 7.5 Hz, Ar–O–(C=O)–CH_2–CH_2–CH_2, 2H) ppm. ^{13}C NMR (125 MHz, $CDCl_3$) δ 172.0, 157.2, 151.1, 148.6, 144.7, 139.9, 136.79, 135.76, 132.6, 132.5, 129.8, 129.0, 128.3, 127.6, 122.3, 121.6, 118.8, 118.4, 114.4, 111.0, 34.2, 33.7, 28.8, 28.6, 28.3, 24.7 ppm.

CBOCO2SBA(CN)

^{1}H NMR (500 MHz, $CDCl_3$) δ 8.51 (s, Ar–N=C*H*, 1H), 8.01 (d, *J* = 8.0 Hz, Ar–*H*, 2H), 7.77 (d, *J* = 8.5 Hz, Ar–*H*, 2H), 7.73 (d, *J* = 8.0 Hz, Ar–*H*, 2H), 7.65 (d, *J* = 8.0 Hz, Ar–*H*, 2H), 7.60 (d, *J* = 8.0 Hz, Ar–*H*, 2H), 7.49 (d, *J* = 8.5 Hz, Ar–*H*, 2H), 7.23 (d, *J* = 8.5 Hz, Ar–*H*, 2H), 7.22 (d, *J* = 8.5 Hz, Ar–*H*, 2H), 3.32 (t, *J* = 7.3 Hz, Ar–S–

CH_2, 2H), 2.93 (t, *J* = 7.3 Hz, Ar–O–(C=O)–CH_2, 2H) ppm. ^{13}C NMR (125 MHz, $CDCl_3$) δ 170.1, 157.8, 150.9, 149.7, 144.6, 139.7, 137.0, 133.4, 132.64, 132.55, 131.5, 129.1, 128.4, 127.7, 122.2, 121.8, 118.8, 118.4, 114.5, 111.1, 34.5, 29.5 ppm.

CBOCO4SBA(CN)

^{1}H NMR (400 MHz, $CDCl_3$) δ 8.50 (s, Ar–N=C*H*, 1H), 8.00 (d, *J* = 8.4 Hz, Ar–*H*, 2H), 7.76 (d, *J* = 8.0 Hz, Ar–*H*, 2H), 7.72 (d, *J* = 8.0 Hz, Ar–*H*, 2H), 7.65 (d, *J* = 8.0 Hz, Ar–*H*, 2H), 7.58 (d, *J* = 8.4 Hz, Ar–*H*, 2H), 7.40 (d, *J* = 8.0 Hz, Ar–*H*, 2H), 7.21 (d, *J* = 8.0 Hz, Ar–*H*, 2H), 7.17 (d, *J* = 8.4 Hz, Ar–*H*, 2H), 3.02 (t, *J* = 7.0 Hz, Ar–S–CH_2, 2H), 2.63 (t, *J* = 7.2 Hz, Ar–O–(C=O)–CH_2, 2H), 1.94 (tt, *J* = 7.4 and 7.2 Hz, Ar–O–(C=O)–CH_2–CH_2, 2H), 1.81 (tt, *J* = 7.4 and 7.0 Hz, Ar–S–CH_2–CH_2, 2H) ppm. ^{13}C NMR (100 MHz, $CDCl_3$) δ 171.7, 157.3, 151.0, 148.8, 144.7, 139.9, 136.8, 135.2, 132.6, 132.5, 130.1, 129.0, 128.3, 127.6, 122.3, 121.7, 118.8, 118.4, 114.4, 111.0, 33.8, 33.5, 28.4, 23.9 ppm.

CBOCO8SBA(CN)

^{1}H NMR (500 MHz, $CDCl_3$) δ 8.51 (s, Ar–N=CH–Ar, 1H), 7.99 (d, *J* = 8.0 Hz, Ar–H, 2H), 7.75 (d, *J* = 8.0 Hz, Ar–H, 2H), 7.72 (d, *J* = 8.5 Hz, Ar–H, 2H), 7.65 (d, *J* = 8.5 Hz, Ar–H, 2H), 7.58 (d, *J* = 8.5 Hz, Ar–H, 2H), 7.36 (d, *J* = 8.5 Hz, Ar–H, 2H), 7.20 (d, *J* = 8.5 Hz, Ar–H, 2H), 7.19 (d, *J* = 8.5 Hz, Ar–H, 2H), 2.95 (t, *J* = 7.5 Hz, Ar–S–CH_2, 2H), 2.59 (t, *J* = 7.5 Hz, Ar–O–(C=O)–CH_2, 2H), 1.77 (tt, *J* = 7.4 and 7.5 Hz, Ar–O–(C=O)–CH_2–CH_2, 2H), 1.68 (tt, *J* = 7.5 and 7.5 Hz, Ar–S–CH_2–CH_2, 2H), 1.49–1.32 (m, Ar–(C=O)–O–CH_2–CH_2–CH_2–CH_2–CH_2–CH_2, 8H) ppm. ^{13}C NMR (125 MHz, $CDCl_3$) δ 172.2, 157.1, 151.2, 148.5, 144.7, 139.9, 136.8, 136.0, 132.6, 132.5, 129.6, 128.3, 127.6, 122.3, 121.6, 118.8, 118.4, 114.3, 111.0, 34.3, 33.7, 29.1, 28.97, 28.94, 28.7, 24.8 ppm.

CBOCO10SBA(CN)

^{1}H NMR (500 MHz, $CDCl_3$) δ 8.51 (s, Ar–N=CH–Ar, 1H), 8.00 (d, *J* = 8.5 Hz, Ar–H, 2H), 7.75 (d, *J* = 8.5 Hz, Ar–H, 2H), 7.72 (d, *J* = 8.0 Hz, Ar–H, 2H), 7.65 (d, *J* = 8.0 Hz, Ar–H, 2H), 7.59 (d, *J* = 8.0 Hz, Ar–H, 2H), 7.36 (d, *J* = 8.0 Hz, Ar–H, 2H), 7.20 (d, *J* = 8.5 Hz, Ar–H, 4H), 2.94 (t, *J* = 7.3 Hz, Ar–S–CH_2, 2H), 2.59 (t, *J* = 7.5 Hz, Ar–O–(C=O)–CH_2, 2H), 1.77 (tt, *J* = 7.4 and 7.5 Hz, Ar–O–(C=O)–CH_2–CH_2, 2H), 1.67 (tt, *J* = 7.5 and 7.5 Hz, Ar–S–CH_2–CH_2, 2H), 1.50–1.39 (m, Ar–(C=O)–O–CH_2–CH_2–CH_2 and Ar–S–CH_2–CH_2–CH_2, 4H), 1.36–1.23 (m, Ar–(C=O)–O–CH_2–CH_2–CH_2–CH_2–CH_2–CH_2–CH_2, 8H) ppm. ^{13}C NMR (125 MHz, $CDCl_3$) δ 172.2, 157.1,

151.2, 148.5, 144.8, 139.9, 136.0, 132.6, 132.5, 129.6, 129.0, 128.3, 127.6, 122.3, 121.6, 118.8, 118.4, 114.3, 111.0, 34.4, 33.7, 29.4, 29.3, 29.2, 29.10, 29.05×2, 28.8, 24.9 ppm.
